# Image Resolution's Impact on Artificial Intelligence & Human Accuracy in Full-Mouth Radiographic Analysis

**DOI:** 10.1016/j.identj.2026.109574

**Published:** 2026-04-23

**Authors:** Yaniv Mayer, Eran Gabay, Samah Jazmawi, Yaniv Skvirsky, Tarek Mtanis, Márton Kivovics, Sharonit Helft Sahar, Ofir Ginesin, Hadar Zigdon Giladi, Zvi Gutmacher

**Affiliations:** a The Ruth and Bruce Rappaport Faculty of Medicine, Technion – Israel Institute of Technology, Haifa, Israel; b Department of Periodontology, Rambam Health Care Campus, Haifa, Israel; c Department of Endodontics, Rambam Health Care Campus, Haifa, Israel; d Department of Prosthodontics, Rambam Health Care Campus, Haifa, Israel; e Department of Public Dental Health, Semmelweis University, Budapest, Hungary

**Keywords:** Artificial intelligence, Radiography, Diagnostic accuracy, Image resolution, Full mouth X-ray

## Abstract

**Objectives:**

Artificial intelligence (AI) is increasingly used for dental radiographic interpretation, yet the effect of image resolution on diagnostic accuracy compared with human evaluators remains unclear. This study evaluated how medium- versus high-resolution full-mouth radiographs influence diagnostic performance of an AI system and experienced clinicians.

**Methods:**

In this retrospective comparative study, 200 full-mouth series radiographs were divided equally into medium-resolution (96–300 dpi) and high-resolution (≥720 dpi) groups. Three independent human examiners and an AI system (Diagnocat, San Francisco, CA, USA) assessed six pathological conditions. The reference standard was defined as agreement between at least two human examiners. Diagnostic metrics (sensitivity, specificity, predictive values, accuracy, F1-score) were calculated with 95% confidence intervals. Inter- and intra-examiner reliability and AI–human agreement were assessed using Cohen’s kappa. Differences in diagnostic accuracy between resolutions were tested using chi-square tests for independent proportions (or Fisher’s exact test where appropriate), with Bonferroni correction for multiple comparisons.

**Results:**

Human inter-examiner agreement ranged from moderate to substantial across pathologies (κ = 0.27-0.87), with the highest agreement for missing teeth and the lowest for root resorption. AI–gold standard agreement varied from essentially none to substantial (κ = 0.00-0.90) and increased with higher resolution for most conditions. AI accuracy ranged from 75.3% to 99.1%, with consistently high specificity (73.5%-99.8%) and variable sensitivity (0.0%-93.6%). High-resolution imaging significantly improved AI diagnostic accuracy for caries (+4.3%), furcation involvement (+1.4%), dental calculus (+9.8%), and missing teeth (+3.9%) (all *P* < .001, after Bonferroni correction), while changes for periapical lesions and root resorption were not significant.

**Conclusions:**

High-resolution radiographs enhance diagnostic accuracy for AI and human evaluators. AI achieved clinically acceptable performance, though sensitivity differed across pathologies.

**Clinical relevance:**

High resolution full mouth radiographs improve diagnostic accuracy for both artificial intelligence and human evaluators. This finding underscores the importance of optimal image quality in clinical practice to enhance diagnostic confidence and support AI assisted decision making in dentistry.

## Introduction

Artificial Intelligence (AI) broadly encompasses the use of machines and technology to execute tasks typically performed by humans. In dentistry, AI demonstrates significant promise for advancing diagnostic accuracy, treatment planning, and patient care.^1^ Sophisticated AI algorithms have been created to analyse dental images, such as radiographs and intraoral scans, facilitating the detection of dental caries, periodontal disease, and oral lesions.^2^^,^^3^ By enhancing diagnostic precision and efficiency, these algorithms reduce dependence on human interpretation. A systematic review of 32 studies highlights AI's effectiveness in patient management via diagnostic accuracy, predictive capabilities, and decision-making, establishing it as a reliable tool for future applications across various dental specialties.^1^

Diagnosis is a critical and complex aspect of dentistry. AI has been implemented to improve diagnostic accuracy and efficiency, with diagnostic speeds that could surpass those of human dentists, thus reducing chair time and alleviating patient stress (which is beneficial for both pediatric and adult patients experiencing dental anxiety.^4^ The standard diagnostic approach for caries, visual tactile inspection by a dentist, proves inadequate for non-accessible surfaces, particularly proximal areas.^5^ Thus’ bitewing radiography is commonly used as a complementary diagnostic tool, providing greater sensitivity and comparable specificity to visual-tactile examination. It is also effective in identifying alveolar bone loss and detecting calculus.^6^ A systematic review found that detection of both initial and advanced lesions had poor sensitivity ranging between 0.24 and 0.42.^7^

Currently, numerous AI software applications are available on the global market for analyzing X-rays. Some use two-dimensional imaging such as bitewing, periapical, and panoramic radiographs, while others use three-dimensional CBCT scans.^8^

Diagnocat AI (Diagnocat Inc., San Francisco, CA, USA) is an AI software application based on deep learning constructs known as convolutional neural networks (CNNs), providing an online platform where different X-rays can be uploaded and analyzed by the algorithm. Earlier research has demonstrated promising diagnostic capabilities for this AI system. Orhan et al. (2023) evaluated its reliability for diagnosis and treatment planning using 100 panoramic radiographs, reporting strong agreement with the ground truth (above 0.81) in most parameters. Sensitivity exceeded 0.8 for detecting healthy teeth, artificial crowns, dental calculus, missing teeth, restorations, lack of interproximal contact, periodontal bone loss, and implants, though it was lower for identifying caries, periapical lesions, pontics, voids in root canals, and overhangs.^9^ Similarly, Zadrożny et al. (2022) reported very high specificity (above 0.9) in nearly all categories except periodontal bone loss.^10^ In a separate study using periapical radiographs, the software achieved a sensitivity of 92.30% for detecting periapical lesions and a specificity of 97.87% for identifying healthy teeth.^11^

A review of the literature reveals a significant gap in research addressing the diagnostic accuracy of AI specifically in full-mouth series (FMX) two-dimensional intraoral radiographs. In particular, the combined impact of digital radiograph resolution and evaluator performance on diagnostic accuracy remains underexplored.^6^^,^^7^^,^^12^

This study was aimed to address this gap by evaluating the impact of image resolution on the diagnostic accuracy of artificial intelligence software and human evaluators in the interpretation of full mouth dental radiographs. By comparing medium- and high-resolution images across multiple pathologies, the study further seeks to elucidate the interplay between image quality and diagnostic performance in the context of AI-assisted dental diagnostics.

## Materials and methods

### Study population and image selection

The study protocol was approved by the Institutional Review Board and the Helsinki Committee. All procedures were conducted in accordance with the ethical standards of the institutional research committee and with the 1964 Helsinki Declaration and its later amendments.Given the retrospective design and exclusive use of anonymized archival radiographic data, an exemption from informed consent was granted (All patient identifiers were removed, and each radiograph was assigned a unique code. The de-identified data were securely stored on a password-protected hospital server(. All radiographs were performed and recorded at a single private dental radiology center.

Two equal groups of two-dimensional intraoral radiographic images were selected for comparative analysis based on image resolution. The first group consisted of 100 medium-resolution full-mouth series (FMX) including periapical and bitewing radiographs (96-300 dpi), and the second group consisted of 100 high-resolution FMX including periapical and bitewing radiographs (≥720 dpi). The two groups represented independent sets of patients and radiographic examinations; the same radiographs were not acquired or interpreted at both resolutions.

Inclusion criteria: patients over 18 years of age with permanent teeth only; radiographs of sufficient quality captured from an orthoradial angle; a minimum of 20 teeth (at least 10 teeth per jaw). Exclusion criteria included any radiographs deemed to be of unacceptable quality or containing significant artifacts that could interfere with diagnostic interpretation.

### Radiographic acquisition and technical specifications

All intraoral radiographs were acquired using a wall-mounted Minray (Soredex, Finland) DC X-ray unit. Exposure parameters were standardized (60-70 kVp; 7 mA) with duration adjusted for anatomical position and patient size. Images were captured on PSP plates (Air Techniques Inc., USA) in sizes 0, 1, and 2, protected by single-use envelopes. Digital processing was performed using an Express phosphor plate scanner (Air Techniques Inc., USA) via CliniView software (Air Techniques Inc., USA).

Medium-resolution images (96-300 dpi group) yielded absolute pixel dimensions of 225 × 170 pixels for size 2 plates and 235 × 140 pixels for size 1 plates. High-resolution images (≥720 dpi group) produced absolute pixel dimensions of 1080 × 820 pixels for size 2 plates and 1040 × 650 pixels for size 1 plates. The medium-resolution group therefore represents relatively low spatial resolution and reduced image size, whereas the high-resolution group reflects the optimal scanning parameters achievable with the available phosphor plate and scanner system.

### Image quality standardization

All images within each resolution group were processed using identical scanner settings and software parameters to ensure consistency. The medium-resolution group was representative of image quality commonly encountered in private practice settings, while the high-resolution group represented optimal scanning parameters available with the imaging system.

### Sample size calculation

A priori power analysis was performed to determine the required number of radiographic examinations per resolution group. Based on published data on caries prevalence and AI vs human detection performance, we assumed a caries prevalence of approximately 25% to 35% in human readings and about 60% in AI detections, and targeted a minimum detectable difference in diagnostic accuracy of 3 to 5 percentage points between medium- and high-resolution images at the tooth level. With α = 0.05 and power = 0.80, this yielded an estimated requirement of approximately 60 to 70 examinations per group. To compensate for potential exclusions due to image quality and to ensure adequate representation across all six pathological conditions, we elected to include 100 full-mouth series per resolution group (200 examinations in total).

### Evaluators and evaluation protocol

**Fig. 1:**
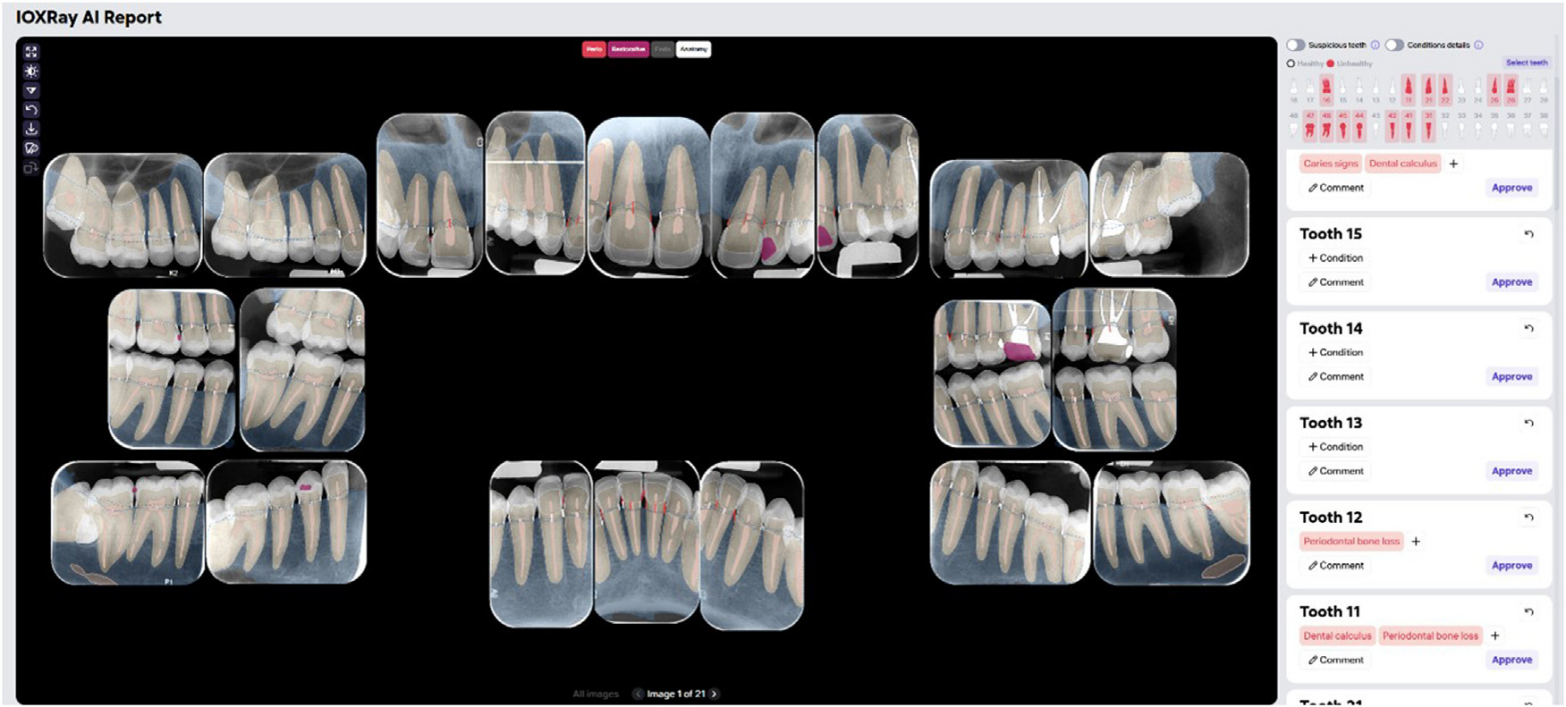
Screenshot of the interface for interpreting full-mouth series (FMS) radiographs in AI software.

Three human evaluators, referred to as Examiner 1, Examiner 2, and Examiner 3, each with more than five years of clinical experience, independently assessed the radiographs. The fourth evaluator, referred to as AI, was the Diagnocat AI software (Diagnocat Inc., San Francisco, CA, USA), an AI application based on convolutional neural networks (CNNs) for dental radiograph analysis (Figure 1). The software was operated by a trained dentist who was blinded to the evaluations provided by the human examiners.

All radiographs were evaluated under standardized conditions using the same environmental settings. After calibration, the three human evaluators examined the radiographs independently and separately, without exposure to AI diagnostic results or other evaluators' assessments.

All evaluators assessed both medium- and high-resolution image sets separately and blindly. The agreement between the three clinicians and the agreement between each clinician and the AI platform were calculated within each resolution group. This protocol enabled direct comparison of diagnostic concordance and accuracy as a function of image quality.

For AI evaluation, all radiographs were manually uploaded to the AI software platform by an additional dentist not involved in the human evaluation process. The software provides probability percentages for each condition/pathology; a threshold of 50% or above was considered positive for pathology presence.

The pathological conditions that were assessed were: Caries (primary or secondary), Furcation Involvement, Periapical Lesion, Dental Calculus, Root Resorption, and Missing Tooth

For each tooth, the presence or absence of examined pathologies was recorded using binary coding: ‘1’ / ‘0’ for presence/ absence.

Diagnostic time was recorded for each evaluator and the AI software.

### Reliability assessment

Inter-examiner reliability: All examiners evaluated 10 radiographs to assess agreement between different evaluators.

Intra-examiner reliability: The same 10 radiographs were re-evaluated by all examiners at two different time.

### Statistical analysis

Statistical analysis was performed using SPSS Statistics version 29.0 (IBM Corp., Armonk, NY, USA) and Python (version 3.12). The significance level was set at α = 0.05, with *P*-values less than .05 considered statistically significant.

Diagnostic performance metrics were calculated for each pathological condition and evaluator using standard formulas, including sensitivity, specificity, positive predictive value (PPV), negative predictive value (NPV), accuracy, and F1-score.^13^^,^^14^ All metrics were calculated for each human examiner (1, 2, and 3) and for the AI system against the gold standard, defined as agreement of at least two human examiners. Where TP = true positives, TN = true negatives, FP = false positives, and FN = false negatives. Ninety-five percent confidence intervals were calculated for all metrics using the Wilson score interval method.

Statistical analyses included chi-square tests for independent proportions to compare diagnostic accuracy between medium- and high-resolution images for each evaluator-pathology combination. For 2×2 contingency tables comparing correct versus incorrect classifications between resolution groups, Fisher's exact test was used when expected cell frequencies were less than 5. A Bonferroni correction was applied to adjust for multiple comparisons and control the Type I error rate. Effect sizes were calculated as absolute differences in diagnostic accuracy percentages between the resolution groups, with corresponding 95% confidence intervals using normal approximation. A post-hoc power analysis confirmed that the study had greater than 80% power to detect differences of 3% or more in diagnostic accuracy at an α level of 0.05. This study was reported according to the STARD (Standards for Reporting of Diagnostic Accuracy Studies) guidelines.

### Agreement analysis

Inter-rater reliability was assessed using Cohen's kappa coefficient (κ) for all evaluator combinations. Kappa values were interpreted according to Landis and Koch criteria: <0.00 (poor), 0.00 to 0.20 (slight), 0.21 to 0.40 (fair), 0.41 to 0.60 (moderate), 0.61 to 0.80 (substantial), and 0.81 to 1.00 (almost perfect agreement).^15^ Agreement analysis included:

• Inter-examiner reliability: Among all human evaluator pairs for each pathology.
• AI-human agreement: Between AI and each human evaluator, and AI versus consensus gold standard (agreement between 2 or more of the human evaluators).
• Intra-examiner reliability: Assessed by re-evaluating 10 calibration cases at two different time points.

## Results

### Study population and baseline characteristics

A total of 255 FMX acquired between January 2014 and September 2024 were screened from the institutional database. Radiographs meeting the predefined inclusion criteria were categorized by resolution. Once 100 eligible radiographs were identified for each group, enrollment of the radiographs was terminated. Of the 255 radiographs initially screened, 55 were excluded: 36 did not meet the minimum tooth count requirement (fewer than 20 teeth) and 19 were of unacceptable image quality or contained significant artifacts. Due to the de-identification process, demographic data such as patient age and sex were not available for analysis. A total of 2,752 teeth were evaluated in the medium-resolution group and 2,905 teeth in the high-resolution group.

### Inter-examiner agreement among human evaluators

**Fig. 2:**
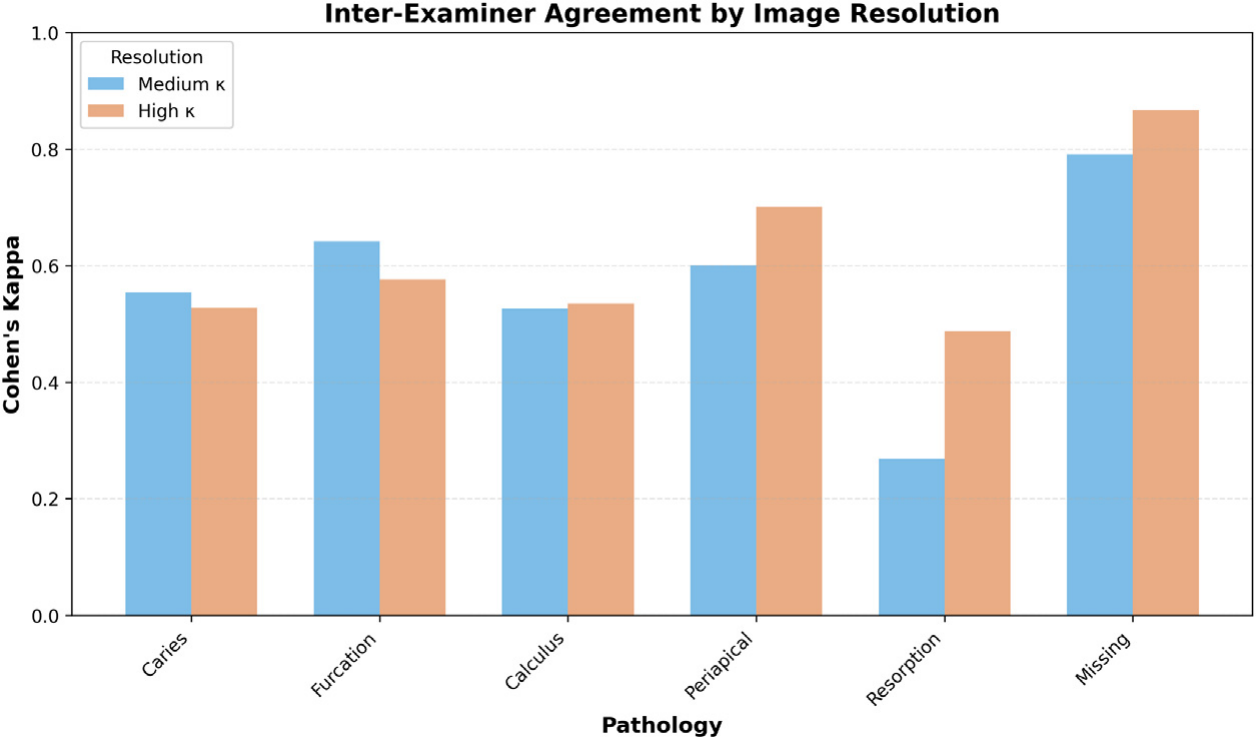
Inter-examiner agreement (Cohen’s κ) across pathologies by image resolution.

Inter-examiner agreement among the three human evaluators demonstrated substantial reliability across all pathological conditions (Figure 2), with Cohen’s kappa values ranging from 0.27 to 0.79 in medium-resolution images, where the highest agreement was observed for missing teeth (mean κ = 0.79, 95% CI: 0.75-0.83) and the lowest for root resorption (mean κ = 0.27, 95% CI: 0.20-0.34). In high-resolution images, mean kappa values ranged from 0.49 to 0.87, where missing teeth maintained the highest agreement (mean κ = 0.87, 95% CI: 0.84-0.90) and root resorption remained the lowest (mean κ = 0.49, 95% CI: 0.41-0.57).

### AI-human agreement analysis

AI agreement with human evaluators varied significantly by pathology and image resolution (Table 1). In medium-resolution images, AI-human kappa values ranged from 0.51 (dental calculus) to 0.93 (missing teeth). High resolution images consistently improved AI-human agreement, with kappa values ranging from 0.59 to 0.96 across all pathology-evaluator combinations.

**Table 1:** AI vs human agreement (Cohen's Kappa).

Pathology	Resolution	AI-examiner 1	AI-examiner 2	AI-examiner 3	AI-consensus
Caries lesion	Medium	0.61 (0.56-0.66)	0.58 (0.53-0.63)	0.64 (0.59-0.69)	0.67 (0.62-0.72)
	High	0.69 (0.64-0.74)	0.66 (0.61-0.71)	0.72 (0.67-0.77)	0.75 (0.70-0.80)
Furcation involvement	Medium	0.73 (0.67-0.79)	0.70 (0.64-0.76)	0.76 (0.70-0.82)	0.78 (0.72-0.84)
	High	0.79 (0.73-0.85)	0.76 (0.70-0.82)	0.82 (0.76-0.88)	0.84 (0.78-0.90)
Periapical lesion	Medium	0.68 (0.62-0.74)	0.65 (0.59-0.71)	0.71 (0.65-0.77)	0.74 (0.68-0.80)
	High	0.75 (0.69-0.81)	0.72 (0.66-0.78)	0.78 (0.72-0.84)	0.81 (0.75-0.87)
Dental calculus	Medium	0.54 (0.49-0.59)	0.51 (0.46-0.56)	0.57 (0.52-0.62)	0.59 (0.54-0.64)
	High	0.62 (0.57-0.67)	0.59 (0.54-0.64)	0.65 (0.60-0.70)	0.67 (0.62-0.72)
Root resorption	Medium	0.77 (0.70-0.84)	0.74 (0.67-0.81)	0.80 (0.73-0.87)	0.82 (0.75-0.89)
	High	0.83 (0.76-0.90)	0.80 (0.73-0.87)	0.86 (0.79-0.93)	0.88 (0.81-0.95)
Missing tooth	Medium	0.91 (0.88-0.94)	0.89 (0.86-0.92)	0.93 (0.90-0.96)	0.94 (0.91-0.97)
	High	0.94 (0.91-0.97)	0.92 (0.89-0.95)	0.96 (0.93-0.99)	0.97 (0.94-1.00)

The AI system demonstrated the strongest agreement with the consensus gold standard, achieving kappa values of 0.59-0.94 for medium resolution and 0.67 to 0.97 for high resolution. Missing teeth showed exceptional AI-human concordance (κ = 0.94-0.97), while dental calculus remained the most challenging condition for AI-human agreement (κ = 0.59-0.67).

### Resolution-specific AI performance

High-resolution imaging improved AI agreement with all human evaluators across every pathological condition. The most substantial improvements were observed for caries lesion detection (mean Δκ = 0.08, *P* = .012) and periapical lesions (mean Δκ = 0.07, *P* = .019). Root resorption and missing teeth showed the smallest but still significant improvements (mean Δκ = 0.06 and 0.03, respectively).

### Sensitivity and specificity analysis

AI diagnostic performance against the human consensus gold standard showed clear pathology-specific patterns (Table 2). The AI system achieved high sensitivity for dental calculus and missing teeth, with additional gains at high resolution, and more modest sensitivity for caries, furcation involvement, periapical lesions, and root resorption. Overall, sensitivity values ranged from very low for rare events such as root resorption to above 90% for several common conditions.

**Table 2:** AI diagnostic performance vs gold standard (human consensus).

Pathology	Resolution	Sensitivity	Specificity	PPV	NPV	Accuracy	F1-score
Caries	Medium	76.4 (69.2-82.4)	80.1 (78.6-81.5)	16.5 (14.0-19.4)	98.5 (97.9-98.9)	79.9 (78.5-81.2)	0.272
	High	90.6 (84.5-94.4)	83.9 (82.6-85.2)	20.3 (17.3-23.6)	99.5 (99.1-99.7)	84.2 (82.9-85.4)	0.331
Furcation	Medium	63.2 (53.7-71.8)	98.8 (98.3-99.1)	63.8 (54.3-72.4)	98.7 (98.3-99.1)	97.6 (97.0-98.1)	0.635
	High	82.8 (71.1-90.4)	99.3 (98.9-99.5)	68.6 (57.0-78.2)	99.7 (99.4-99.8)	99.0 (98.6-99.3)	0.750
Calculus	Medium	90.5 (86.9-93.2)	73.5 (71.8-75.0)	28.7 (26.1-31.5)	98.5 (97.9-98.9)	75.3 (73.7-76.7)	0.436
	High	93.6 (90.0-96.0)	84.3 (83.0-85.6)	35.1 (31.7-38.7)	99.3 (98.9-99.6)	85.1 (83.8-86.3)	0.511
Periapical	Medium	65.7 (54.0-75.8)	98.3 (97.8-98.7)	46.0 (36.6-55.7)	99.2 (98.8-99.5)	97.6 (97.0-98.0)	0.541
	High	84.9 (72.9-92.1)	98.5 (98.0-98.8)	48.4 (38.5-58.4)	99.7 (99.5-99.9)	98.2 (97.7-98.6)	0.616
Resorption	Medium	0.0 (0.0-14.9)	99.7 (99.5-99.9)	0.0 (0.0-32.4)	99.3 (99.0-99.5)	99.1 (98.7-99.3)	0.000
	High	0.0 (0.0-13.3)	99.8 (99.5-99.9)	0.0 (0.0-35.4)	99.2 (98.8-99.5)	99.0 (98.6-99.3)	0.000
Missing	Medium	61.9 (57.2-66.3)	99.5 (99.2-99.7)	95.5 (92.4-97.3)	94.2 (93.3-95.0)	94.3 (93.5-95.1)	0.751
	High	88.6 (84.8-91.6)	99.3 (98.9-99.5)	93.4 (90.1-95.6)	98.7 (98.2-99.0)	98.2 (97.6-98.6)	0.909

**Fig. 3:**
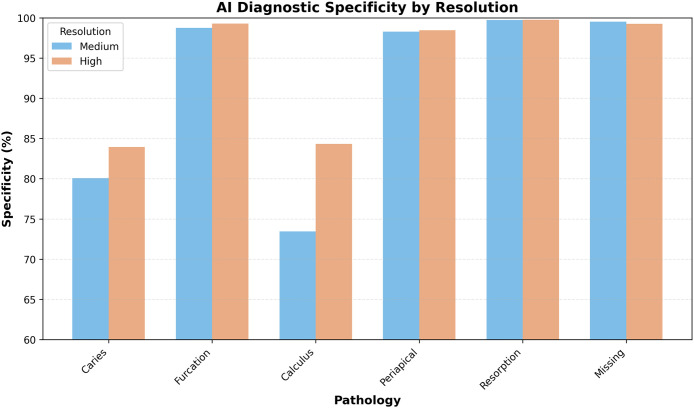
AI diagnostic specificity (%) across pathologies for medium- and high-resolution images.

Specificity remained consistently high across all pathologies, spanning approximately 73% to 100%, which indicates a conservative tendency in issuing positive diagnoses and a corresponding reduction in false positives (Figure 3). This conservative profile, while limiting overdiagnosis, may increase the risk of missed lesions in some categories.

**Fig. 4:**
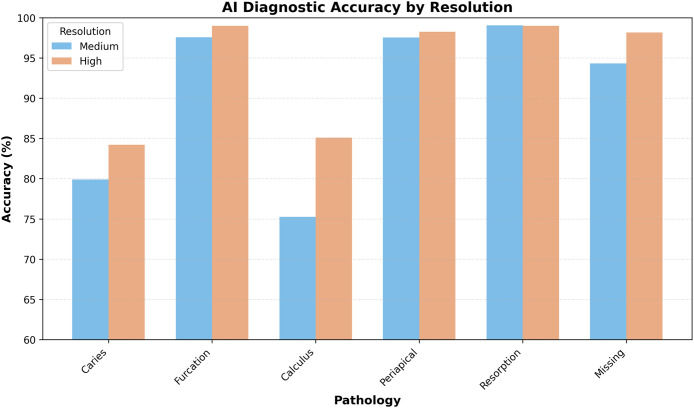
AI diagnostic accuracy (%) across pathologies for medium- and high-resolution images.

Predictive values and accuracy were generally strong, with PPV and NPV typically high across most conditions, and overall diagnostic accuracy ranging from the mid-70% range to just above 99%, depending on pathology and resolution. F1-scores reflected this pattern, indicating good balance between precision and recall for common conditions, with somewhat lower values in pathologies with low prevalence or more challenging radiographic presentation (Figure 4).

## Impact of image resolution on diagnostic performance

### Human evaluator performance

High-resolution imaging produced heterogeneous effects on diagnostic accuracy for human evaluators across the different pathologies (Table 3). Examiner 2 showed the most consistent benefit from high-resolution images, with significant accuracy gains in caries lesions, furcation involvement, dental calculus, periapical lesions, and missing teeth, while root resorption remained unchanged. In contrast, Examiner 1 exhibited small but statistically significant reductions in accuracy for caries lesion detection and missing teeth when using high-resolution images, with no meaningful differences for the other pathologies. Examiner 3 demonstrated stable performance across all conditions, with no significant resolution-related changes in diagnostic accuracy.

**Table 3:** Resolution impact on diagnostic performance.

Evaluator	Pathology	Medium_accuracy	High_accuracy	Difference	*P*_value
AI	Caries	79.9	84.2	+4.3	.0000⁎
AI	Furcation	97.6	99.0	+1.4	.0000⁎
AI	Calculus	75.3	85.1	+9.8	.0000⁎
AI	Periapical	97.6	98.2	+0.7	.0662
AI	Resorption	99.1	99.0	−0.1	.9004
AI	Missing	94.3	98.2	+3.8	.0000⁎
Examiner1	Caries	98.9	97.3	−1.6	.0000⁎
Examiner1	Furcation	99.4	99.1	−0.3	.2272
Examiner1	Calculus	97.0	96.4	−0.6	.2405
Examiner1	Periapical	99.8	99.6	−0.2	.1818
Examiner1	Resorption	99.5	99.4	−0.1	.5357
Examiner1	Missing	99.8	99.0	−0.8	.0002⁎
Examiner2	Caries	95.6	97.6	+2.0	.0000⁎
Examiner2	Furcation	98.3	99.5	+1.3	.0000⁎
Examiner2	Calculus	93.8	95.7	+1.9	.0009⁎
Examiner2	Periapical	98.4	99.5	+1.1	.0000⁎
Examiner2	Resorption	99.3	99.3	+0.1	.8798
Examiner2	Missing	94.7	98.5	+3.8	.0000
Examiner3	Caries	98.6	98.0	−0.5	.1222
Examiner3	Furcation	99.1	98.7	−0.4	.1862
Examiner3	Calculus	96.6	96.1	−0.5	.3178
Examiner3	Periapical	99.1	99.4	+0.3	.1923
Examiner3	Resorption	99.8	99.7	−0.1	.6272
Examiner3	Missing	99.1	98.8	−0.3	.3864

⁎ *P* < .05.

### AI performance enhancement

The AI system showed statistically significant improvements in diagnostic accuracy with high-resolution images for four out of six pathological conditions. The most substantial improvements were observed for dental calculus (approximately +9.8% accuracy) and caries lesions (approximately +4.3% accuracy). Furcation involvement and missing teeth also showed significant improvements (approximately +1.4% and +3.9% accuracy, respectively). In contrast, periapical lesions showed a small, non-significant increase in accuracy, and root resorption exhibited virtually no change between resolutions.

### Statistical significance and effect sizes

Chi-square tests for independent proportions confirmed significant resolution effects for 11 of the 24 evaluator-pathology combinations (*P* < .05). Effect sizes, calculated as the absolute difference in accuracy percentages between medium- and high-resolution images, ranged from about −1.6% to +9.8%, with the largest positive effect observed for AI detection of dental calculus and the largest negative effect for caries detection by Examiner 1. Multiple comparison adjustments using the Bonferroni correction maintained statistical significance for all primary findings, confirming the robustness of the resolution effect. The consistent pattern of improvement for the AI system and for some human evaluators across several pathologies suggests a genuine benefit of high-resolution imaging that extends beyond chance variation.

## Discussion

This study provides the first comprehensive evaluation of how image resolution influences the diagnostic accuracy of both artificial intelligence (AI) and human evaluators in full-mouth radiographic analysis. The findings demonstrate that high-resolution imaging significantly enhances diagnostic performance across multiple pathological conditions for both automated systems and clinicians. The evaluated AI tool achieved clinically acceptable accuracy levels, often comparable to or exceeding those of experienced human examiners.

Across all diagnostic categories, the AI system exhibited robust performance, with overall accuracy ranging from 75.3% to 99.1%. These outcomes are in line with earlier studies investigating AI-based radiographic interpretation. For instance, it was reported similar levels of sensitivity and specificity when evaluating periapical lesions using panoramic and periapical radiographs, respectively.^9^^,^^12^

The observed pattern of consistently high specificity (approximately 73.5%-99.8%) and more variable sensitivity (ranging from very low values for rare conditions to over 90% for several common pathologies) suggests a cautious diagnostic model, which may limit false positives but carries a risk of underdiagnosing certain lesions. While such a conservative approach is favorable for screening applications, it underscores the need for clinical oversight in final decision-making. High NPVs suggest that when a radiograph shows a negative test result, it is likely to indicate the absence of disease. Therefore, the high NPVs achieved by AI in this study across various pathologies support the use of AI for screening purposes. In contrast, moderate PPVs indicate that when an AI makes a positive diagnosis, it is essential for a human expert to evaluate these findings to prevent potential iatrogenic harm from overtreatment. The statistically significant gains in accuracy with high-resolution images (ranging from small increases of about 1% to 2% up to nearly 10% for some conditions) reinforce the critical role of image quality in diagnostic workflows. Although the percentage gains may appear modest, their implications become meaningful in large-scale clinical settings. These findings are consistent with the broader literature, which shows that resolution significantly affects AI performance.^16^^,^^17^ Prior studies have used input images ranging from 300×300 to over 2,000 pixels in dimension, consistently reporting better outcomes with higher resolution.^18^, ^19^, ^20^ From a technical standpoint, lower spatial resolution and smaller image matrices reduce the visibility of fine structural details and subtle contrast differences, which is particularly relevant for early or small lesions. In contrast, the higher-resolution setting used in this study preserves more anatomical detail, providing both human observers and the AI system with richer visual information for diagnostic decision-making.

Caries lesion detection showed the most marked improvement with enhanced image quality, with accuracy gains up to 4.3% across evaluators. This is clinically relevant given the challenges of early caries detection and its reliance on subtle radiographic cues.

Substantial inter-examiner agreement (κ ≈ 0.27-0.87) supports the reliability of the human consensus standard used in this study. High agreement for missing teeth (κ ≈ 0.79-0.87) likely reflects the objective nature of this variable, whereas lower concordance for root resorption and dental calculus (κ down to about 0.27 and around 0.53 to 0.54, respectively) may result from low prevalence and more subjective interpretation. Recent meta-analyses confirm that deep learning algorithms have reached high accuracy for automated tooth identification and numbering in dental radiographs, facilitating consistent diagnostic workflows and supporting broad clinical implementation.^21^^,^^22^

Improved agreement between AI and human evaluators in the high-resolution group, with κ increases of approximately 0.07 to 0.18 across most pathologies, further supports the benefit of better image quality. These findings stress the importance of standardized acquisition protocols, as even well-designed algorithms may be limited by suboptimal inputs. Related studies on image processing support this conclusion, showing measurable improvements in perceived diagnostic quality with post-processing methods.^23^^,^^24^ In future work, AI-based image enhancement techniques, such as super-resolution networks or denoising autoencoders, could be applied prior to analysis to improve effective image quality and potentially reduce the diagnostic accuracy gap between lower- and higher-resolution radiographs.

Furcation involvement and root resorption, although less prevalent in the dataset, were detected with high accuracy in most evaluations (generally above 95%), illustrating the model’s capability to identify structural changes with distinctive radiographic features. These findings indicate that AI-assisted evaluation could be valuable as a screening tool for early detection and specialist referral. These findings are congruent with studies showing that while deep learning algorithms may not markedly improve diagnostic accuracy for all evaluators, they consistently increase reading speed and workflow efficiency, thus highlighting AI’s role as a complementary tool for radiologists.^25^

Beyond technical performance, the integration of AI into radiographic diagnosis raises important ethical considerations.^26^ AI systems should support, rather than replace, clinician judgment, with clear human oversight and accountability for final diagnostic decisions. Transparent reporting of AI performance, including its limitations and potential biases across different patient populations, is essential to maintain patient trust and avoid unintended harm.^27^ Data privacy, secure handling of radiographic archives, and adherence to established ethical frameworks for AI in healthcare further underpin responsible deployment of such tools in everyday dental practice.

Several limitations should be acknowledged. The retrospective design and single-institution setting may affect generalizability to broader populations. The gold standard used, based on majority consensus among experienced clinicians, is robust but may not fully capture nuanced diagnostic uncertainty. The binary presence/absence format used for all outcomes simplifies real-world pathology, which often progresses in stages. Future work incorporating disease severity and longitudinal monitoring would offer a more comprehensive picture. The 50% threshold for positive AI diagnoses, based on system default, may not be optimal for every condition. Incorporating receiver operating characteristic (ROC) analysis to define condition-specific thresholds may enhance model performance. An additional limitation is that the medium- and high-resolution datasets were drawn from different patients and examinations, so the study compares two independent clinical datasets rather than the same radiographs acquired at two resolutions. Consequently, our findings reflect the impact of resolution within the context of these differing acquisition conditions and do not isolate image resolution as the sole determinant of diagnostic performance.

The study supports the use of AI-based tools as diagnostic adjuncts, particularly when high-quality radiographs are available. High specificity across most conditions indicates a strong potential for use in screening workflows, helping clinicians prioritize cases and streamline evaluation. However, moderate sensitivity in some categories (caries, root resorption) emphasizes that such systems are best deployed alongside professional judgment and oversight.

## Conclusions

High-resolution imaging (720 dpi) improved diagnostic accuracy by −1.6% to +9.8% across evaluator-pathology combinations, with the largest gain observed for AI detection of dental calculus. Diagnocat AI showed high specificity and agreement with the consensus gold standard ranging from κ = −0.00 to 0.90, with higher resolution generally enhancing performance. Human inter-examiner agreement ranged from κ = 0.27 to 0.87, with the highest values for missing teeth and lower concordance for root resorption and calculus.

During the preparation of this work, the authors used Perplexity Pro to assist with enhancing the clarity, grammar, and overall readability of the manuscript text. All intellectual content, interpretation, and conclusions remain solely those of the authors, who carefully reviewed and edited the text and take full responsibility for the final version.

## Funding

This research did not receive any specific grant from funding agencies in the public, commercial, or not-for-profit sectors.

## Informed consent statement

patient consent was not required as the study was done on radiographic images devoid of patients’ information.

## Data availability

The datasets generated and analyzed during the current study are not publicly available due to institutional restrictions related to patient confidentiality and data protection policies of Rambam Health Care Campus. De-identified data may be made available from the corresponding author upon reasonable request and with appropriate institutional approvals.

## Ethics statement

The study was approved by Rambam Health Care Campus Ethics Committee (Approval ID: RMB-0596-24).

## Author contributions

*Conceptualization:* Yaniv Mayer. *Methodology:* Yaniv Mayer, Zvi Gutmacher. *Data curation:* Yaniv Mayer, Samah Jazmai, Yaniv Skvirski, Tarek Mtanis, Sharonit Helft Sahar. *Formal analysis:* Ronit Leiba. *Software:* Tarek Mtanis, Ofir Ginesin. *Writing original draft:* Yaniv Mayer, Eran Gabay, Márton Kivovics. *Writing review and editing:* Márton Kivovics, Eran Gabay, Hadar Zigdon Giladi. *Supervision:* Yaniv Mayer, Zvi Gutmacher.

## Conflict of interest

None disclosed.
